# The Emerging Threat of *Plasmodium knowlesi* Malaria Infection: A Concept Paper on the Vulnerable Factors in Human

**DOI:** 10.3390/ijerph19074419

**Published:** 2022-04-06

**Authors:** Nurul Athirah Naserrudin, Rozita Hod, Mohammad Saffree Jeffree, Kamruddin Ahmed, Mohd Rohaizat Hassan

**Affiliations:** 1 Department of Community Health, Faculty of Medicine, Universiti Kebangsaan Malaysia, Kuala Lumpur 56000, Malaysia; drathirah85@gmail.com (N.A.N.); rozita.hod@ppukm.ukm.edu.my (R.H.); 2 Borneo Medical and Health Research Centre, Faculty of Medicine and Health Sciences, Universiti Malaysia Sabah, Kota Kinabalu 88400, Malaysia; saffree@ums.edu.my (M.S.J.); ahmed@ums.edu.my (K.A.); 3 Sabah State Health Department, Ministry of Health, Putrajaya 62590, Malaysia; 4 Department of Public Health, Universiti Malaysia Sabah, Kota Kinabalu 88400, Malaysia; 5 Department of Pathobiology and Medical Diagnostics, Faculty of Medicine and Health Sciences, Universiti Malaysia Sabah, Kota Kinabalu 88400, Malaysia

**Keywords:** malaria, *Plasmodium knowlesi*, zoonotic malaria, vulnerability factors, malaria exposure

## Abstract

In South-East Asia (SEA), there has been an increase in the trend of detected and reported cases of *Plasmodium knowlesi* malaria in the last few decades. This higher transmission rate within SEA countries is attributed to the distribution of the *Macaque*, banded leaf monkeys, and *Anopheles* mosquito in this region. This study aims to propose a concept that highlights the integration of vulnerability factors to *P. knowlesi* malaria infection. The relevant literature on the vulnerability factors of *P. knowlesi* was reviewed. Any theories and models that could be integrated to support the factors were also explored throughout this study. Exposure to *P. knowlesi* malaria was found to be influenced by sociodemographic, socioeconomic, environmental, social context, belief, and human behaviour factors. However, these factors were commonly discussed separately in existing studies. For better disease prevention and control, all these factors should be explored collectively, to facilitate research aimed at generating a deeper understanding of the vulnerability factors to *P. knowlesi* malaria from various perspectives, including the genetic, sociodemographic, socioeconomic, environmental, sociocultural beliefs, and human behaviour of the population.

## 1. Research Manuscript Section

### 1.1. Introduction

**Figure 1:**
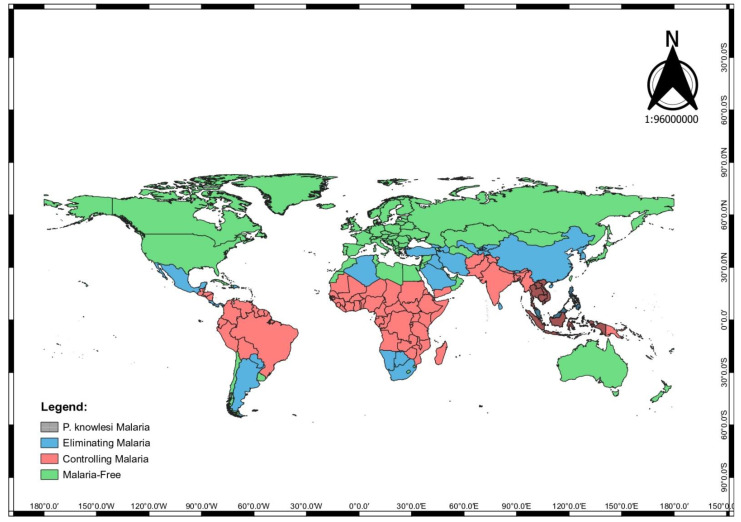
Areas with high risk of *P. knowlesi* malaria infection in the world map.

*Plasmodium knowlesi* (*P. knowlesi*) is a parasite of non-human primates that can cause zoonotic diseases in humans. The epidemiology of *P. knowlesi* malaria is complex as it involves the *Macaque* monkey and banded-leaf monkey (*Presbytis melalophos*) as the natural host, *Anopheles* mosquito as the vector, and human as the host [1,2]. Initial research suggested *P. knowlesi* malaria as a rare infection among humans [1]. However, in 2004, a significant number of human *P. knowlesi* malaria infections were identified in Sarawak, Malaysia [1]. In the last few years, more cases of *P. knowlesi* malaria have been diagnosed in humans, raising the possibility of human-vector-human transmission, especially when the infection was detected peridomestically among household members [2]. Due to the upsurge of cases among humans, *P. knowlesi* has been identified as the fifth species of human malaria [3]. While worldwide, the human malaria cases that are caused by *P. falciparum* and *P. vivax* remain a burden in the African, South-East Asia (SEA), Eastern Mediterranean, and Western Pacific region, the escalating *P. knowlesi* malaria infection in the human population is considered a threat to public health [2]. In 2020, the World Health Organization (WHO) included *P. knowlesi* malaria in the annual World Malaria Report [4]. Despite the success of the elimination programme for human malaria, the rising incidence of *P. knowlesi* malaria among human in the SEA region, is worrying [5] (see Figure 1).

To date, the exponential increase in the incidence of *P. knowlesi* human malaria cases, including asymptomatic cases, have been associated with factors such as ecological changes that are secondary to deforestation, a decrease in the human malaria cases of other species that resulted in reduced relative immunity, increasing awareness among the communities to *P. knowlesi* malaria, and improvement of the diagnostic capacity [2,5]. Before 2008, malaria was traditionally diagnosed using blood smears and conventional light microscopy (LM) [1]. After 2008, Malaysia initiated the polymerase chain reaction (PCR)-based malaria method for *P. knowlesi* diagnosis [5]. Currently, *P. knowlesi* malaria screening is commonly done at Government healthcare facilities, by withdrawing the blood sample from febrile patients [6]. However, conventional diagnostic methods such as blood smears are not effective at diagnosing asymptomatic *P. knowlesi* malaria infection because the level of parasite density is too low to be detected via LM [5]. As a result, the conventional LM method can result in the misdiagnosis of *P. knowlesi* malaria infection. The complexity of the *P. knowlesi* parasite characteristic and features, as well as its similarity with the other *Plasmodium* species such as *P. falciparum* and *P. malariae* can present as a diagnostic challenge [3]. *P. knowlesi* and *P. malariae* share certain similarities and features in the blood stages, such as the presence of band-form trophozoites [3]. Moreover, previous studies showed that *P. knowlesi* mortalities have been misdiagnosed as *P. malariae* infection [7]. On the other hand, the difficulty in identifying the morphology of *P. knowlesi* and *P. falciparum* species arises as both species show double chromatin dot features without the enlargement of infected erythrocytes during their early trophozoite stage [3]. Therefore, the level of parasitaemia is not a sensitive laboratory method for malaria detection.

*P. knowlesi* malaria infection can result in a range of outcomes, from asymptomatic, uncomplicated, complicated, to fatalities. Therefore, it is imperative to design and implement specific and sensitive diagnostic methods [2,5]. In Malaysia, molecular diagnostic screening using PCR has significantly increased the detection of *P. knowlesi* malaria cases, especially in the Malaysian Borneo region [3]. The use of molecular assays i.e., PCR, to detect malarial parasite DNA has created a new window in the diagnostic capability for malaria infection [2]. However, it remains challenging to accurately diagnose *P. knowlesi* in rural healthcare facilities. The mainstay of diagnostic modality in rural clinics is the LM method with a lower reliability rate [5]. Despite the superiority of PCR in providing a more sensitive and specific diagnosis, the service is only available in reference laboratories that are located far from rural areas [8].

While *P. knowlesi* malaria cases have been detected in human populations that are living in Thailand, Cambodia, Myanmar, and Malaysia, the cases were mostly confined to the natural distribution of natural hosts and vectors of *P. knowlesi*. In 2018, 4131 *P. knowlesi* malaria cases were detected in Malaysia as compared to only 376 cases in 2008. The incidence rate was 0.13 per 1000 population in 2018 [5]. Worldwide, the state of Sabah, located in the Malaysian Borneo Island, reported the highest incidence of *P. knowlesi* cases in humans [5]. In addition, recent studies reported cases among returning travellers from countries in the SEA, thereby raising the urgency to instil awareness about personal protective measures and the symptoms of *P. knowlesi* infection among travellers [9].

Apart from the human-vector-human transmission, blood transfusion was also noted to be the cause of *P. knowlesi* malaria infection [10]. Moreover, the outcome was severe malaria in the splenectomised thalassemia patients [10]. Symptomatic *P. knowlesi* cases were found in all age groups [5,6]. Patients experienced nonspecific symptoms such as fever headache, myalgia/arthralgia, and poor food intake [3]. In addition to the symptomatic cases, a proportion of individuals have been diagnosed with asymptomatic infection in recent studies [11,12]. The prevalence of asymptomatic cases may also be under-reported if the density of the parasite is too low to be detected by conventional microscopy [12]. Hence, it can manifest as a “silent” carrier. The asexual life cycle of *P. knowlesi* is short, and a rapid increment of parasitaemia can be achieved within 24 h [3,13], unlike 48 h duration in *P. falciparum, P.vivax,* and *P. ovale* and 72 h in *P. malariae* infection [3,13]. Currently, the most effective first-line treatment for uncomplicated *P. knowlesi* malaria is arthemeter–lumefantrine, which is associated with good tolerability and rapid therapeutic response [2]. The therapeutic effect of conventional anti-malarial medication such as chloroquine and mefloquine against *P. knowlesi* malaria is moderate [5]. Without immediate and appropriate treatment, *P. knowlesi* infection can result in severe outcome including fatalities [7]. However, cerebral malaria is not a feature of *P. knowlesi* malaria infection, unlike *P. falciparum* cases [3]. In addition, the parasite can harbored for a long time in these asymptomatic cases, silently turning into a reservoir. Thus, asymptomatic malaria infection can spark unusual outbreaks and evolve into a hotspot of disease transmission [12]. Meanwhile, in *P. knowlesi* malaria transmission, this question remains, and further studies are further needed as evidence. Current and future research must progress synergistically with the dynamic changes of this zoonotic *P. knowlesi* malaria infection. Despite a better understanding of the life cycle of *P. knowlesi* malaria and the potential threat of asymptomatic *P. knowlesi* to public health, studies investigating new *P. knowlesi* malaria therapeutic drugs should continue [2]. Although currently *P. knowlesi* malaria treatments are manageable with antimalarials, the detection of new *Plasmodium* species strains in Myanmar, Cambodia, and Thailand which are resistant to drugs, shows that studies should continue to investigate new antimalarial drugs [2].

In view of the emerging threat of this zoonotic infection, it is vital to determine the vulnerability factors to *P. knowlesi* to reduce the incidence of infection among humans, including asymptomatic infection. Currently, the majority of existing evidence of behavior and malaria exposure were obtained from quantitative research and less so from qualitative research [14,15]. The social context and environmental challenges have been reported as evidence that affects *P. knowlesi* malaria control [15]. With the change in the epidemiology of *P. knowlesi* infection, the existing guidelines must be updated accordingly, to enable a more robust assessment of the infection. Hence this study aimed to propose a concept that would include possible vulnerability factors of humans to *P. knowlesi* malaria and how these factors affect the disease severity. The identification of these factors can facilitate better *P. knowlesi* malaria prevention and control strategies. The relevant existing literature that was published on the vulnerability factors of *P. knowlesi* malaria infection in humans was reviewed. In addition, theories and models that could be integrated into future studies were also explored to strengthen the findings of this study. The data were extracted into Microsoft Excel, and narrative data analysis was performed.

### 1.2. Vulnerability Factors of P. knowlesi Infection in Human

To reduce *P. knowlesi* malaria infection, it is imperative to identify all the vulnerability determinants of humans to the infection. The vulnerability factors of *P. knowlesi* are complex as they are made up of a combination of a change in the exposure and sensitivity of humans to the parasite and environment [5]. The transmission is mainly determined by the presence of the *Macaque* monkey, the *Anopheles* mosquito, and a suitable environment [16]. Large foci of humans were diagnosed with the infection in 2004 in the area of Kapit, Sarawak, on the Borneo Island of Malaysia [3]. Since then, the increased threat of *P. knowlesi* human infection has led to its inclusion in the World Malaria Report [4].

### 1.3. Human Genetic

Human genetics are crucial in determining the susceptibility of an individual to the infection [17]. The understanding of the genetic mechanism of resistance or increased susceptibility to infection can provide new approaches to the control and management of the disease [18]. According to Amir et al., the high genetic diversity and selection pressure of various proteins that are found in *P. knowlesi* from human and monkey samples, can contribute to the polymorphisms in the immune response [2]. Generally, genetic disorders of the red blood cells that involve changes to haemoglobin, cell membrane, or metabolic pathways have been linked with changes in malaria resistance [19]. In the case of *P. knowlesi* malaria, such blood disorders include glucose 6-phosphate dehydrogenase (G6PD) enzyme deficiency that appeared to confer a certain degree of protection against *P. knowlesi* malaria, as shown in several studies that were conducted among the human population of the Northern Borneon Island [2,6]. G6PD enzyme deficiency is associated with a decreased risk of symptom manifestation and adverse outcomes in the *P. knowlesi* cases. Such features are likely related to the long-term selection pressure from previous exposure to human malaria infection [6].

### 1.4. Human Immune Response

The host immune response to *P. knowlesi* exposure may also influence the clinical outcomes of the infection in terms of disease severity, i.e., uncomplicated and complicated outcomes [20]. For instance, the development of immunity after frequent exposure and living in the same villages that are known to be *P. knowlesi*-exposed areas for more than six months were found to provide protective benefits to the communities there [6]. Apart from that, the outcome of the disease may vary according to the individual’s cytokine levels. Cytokine is a human protein that functions as an acute mediator of inflammation [21]. The cytokine theory postulates that parasites that are sequestered in the local blood vessels during malaria infection may produce higher than average cytokine concentrations at the local site compared to the general circulation [21]. Furthermore, different outcomes were detected in *P. knowlesi* cases based on differences in the anti- and pro-inflammatory cytokine levels [20]. Therefore, the measure of disease severity using cytokine levels can be a good indicator of the host immune response against the infectious agent [20].

At present, it is argued that both the parasite and host factors can influence the outcome of the infection as to whether the infection remains asymptomatic or becomes symptomatic and potentially life-threatening [20,21,22]. Moreover, with a significant increase in the asymptomatic *P. knowlesi* cases, it is vital to understand the disease progression. Lastly, it was also found that malaria outbreaks commonly occur in the areas that experience the change in climate or other conditions that favour the transmission, such as in areas where people have little or no immunity against malaria [23]. Therefore, the combination of both climate change and the absence of immunity may further alter the transmission pattern of the pathogen, thus resulting in the potential emergence of malaria in areas where it had not previously been present or reported [23].

### 1.5. Sociodemographic

From the perspective of sociodemographic characteristics, the infection of *P. knowlesi* malaria is strongly influenced by gender and age [5,6]. The majority of the studies agreed that the incidence of symptomatic *P. knowlesi* cases are higher among males due to the nature of their work as agricultural workers, planters, and farmers, that expose them to mosquito bites [5,6]. However, based on *P. knowlesi* malaria seroprevalence studies, the risk of being infected by malaria is equal for both genders [24]. A higher proportion of asymptomatic *P. knowlesi* infections was detected among household members, including females and children [24].

Previous studies have proven that adults are at a higher risk of *P. knowlesi* malaria infection as they showed a higher level of parasitaemia that would increase the severity of the infection. Children were mainly found to be asymptomatic [11]. While in children, anaemia and thrombocytopenia were common findings at hospital admission and during stay [8]. However, the presence of these asymptomatic *P. knowlesi* cases showed that the true incidence of the infection can be under-reported [5].

### 1.6. Socioeconomic

Poverty, economic growth, inequalities, and disturbance in the conservation of habitats and biodiversity can adversely impact the achievement of sustainable development goals (SDGs) towards malaria elimination by 2030 [4]. Malaria is commonly associated with socioeconomic status (SES) such as education level, occupation, and income [4]. It is widely agreed that exposure to *P. knowlesi* malaria commonly affects individuals in poor economic conditions, such as those who live in the forest or near forest regions [5,16,24]. Communities that are affected by malaria face challenges such as access to medical services and malaria intervention, thus putting them at risk of higher severity and poorer prognosis of the infection [4]. Poverty and the associated conditions such as poor housing and inequity are all linked to a higher risk of malaria and worse consequences of the disease [4,25].

A low SES is linked with a low level of education. Education can influence a ’person’s understanding of malaria-specific preventive measures [25]. The lack of formal education has been shown to increase vulnerability towards malaria infection due to the poor uptake of prevention efforts against malaria [25]. In contrast, an adequate education is associated with a better understanding of malaria transmission and the associated symptoms of the infection [25].

Furthermore, the SES of an individual is also influenced by the type of occupation. The evidence listed oil palm workers, logging farmers, and individuals who work in the forest as more vulnerable to the *P. knowlesi* malaria infection [5,6,16]. Most of these sectors are dominated by males, thus correlating with the previous argument that the prevalence of *P. knowlesi* malaria infection is higher among males than females [2].

Another important determinant of SES is the level of income. Compared to the higher-income group, individuals from the lower-income group were more exposed to the transmission of malaria [4,25]. The limited amount of income may influence the living condition of an individual. For instance, poor living conditions such as wooden houses with cracks (e.g., open eaves and gaps in the wall)—could increase the probability of *P. knowlesi* malaria infection as mosquitoes can enter the house to seek a host [6]. Travelling to and from their workplace in or near the forest by walking, also exposed the individual to the *P. knowlesi* malaria infection [6,16].

### 1.7. Environmental Factor

Next, the incidence of *P. knowlesi* malaria in the human population is also influenced by the surrounding environmental factors [6,16,26]. The vulnerable community often faces a high risk of exposure due to the spillover of the *Macaque* monkey and *Anopheles* mosquitoes to the human settlements as a result of ecological disturbances such as deforestation [16]. Individuals who work or live in or nearby the forest areas are at a higher risk of malaria infection due to the presence of the *Anopheles* mosquitoes and the *Macaque* monkey [16]. Deforestation has continuously increased the probability of *P. knowlesi* malaria transmission [16,26]. As compared with the exposure to *P. falciparum* and *P. vivax* malaria infection, *P. knowlesi* is most affected by deforestation [26]. Deforestation has led to a shift in the distribution of the *Anopheles* vector and natural hosts of malaria infection [16,26]. It is postulated that deforestation destroys the habitat of the long-tailed and pig-tailed macaques, the natural hosts of *P. knowlesi* malaria, causing them to shift from forested areas to farms and semi-urban areas. Due to their blood-sucking nature, the mosquitoes also follow their host and adapt to the forest edge and farms [16].

Beside the above-mentioned environmental factors, certain peri-domestic conditions also increase vulnerability to *P. knowlesi* malaria infection. The *Anopheles* mosquito is an exophagic biter, i.e., it bites outdoors, thus suggesting the presence of peri-domestic transmission among individuals who do not perform forest-related work [6,16]. While the peak biting time for *Anopheles* mosquito from the *Leucosphyrus* and *Barbirostris* groups are between 18:00 to 21:00 h, the *Umbrosus* group was found to bite earlier between 07:00 to 11:00 h [27]. The transmission of malaria due to peri-domestic conditions could be associated with the presence of *Anopheles* mosquito around the house. In a study that was conducted in Sabah, Malaysia, the transmission of malaria could be observed in areas with long grass or near home settlements with the presence of *Macaque* monkeys [6]. In addition, individuals who perform daily routines such as gathering and relaxing outside the house, also predispose them to a higher risk of malaria transmission [16]. Forest workers are another vulnerable group due to their frequent contact with *Macaque* monkeys and *Anopheles* mosquitoes at the workplace [6,28].

### 1.8. Social Context and Belief

Social context and belief also play an important role in malaria exposure [15,28,29]. Various studies in the SEA region that explored the social context in terms of *P. knowlesi* malaria exposure are available. In the Philippines, the community perception of malaria infection influenced their treatment-seeking behaviour and self-efficacy in performing preventive measures [30]. In Indonesia, forest workers showed poor self-efficacy in terms of bednet usage and poor perception of the threat of the disease [28]. The community that was highly exposed to *P. knowlesi* malaria in Sabah, Malaysia was mainly villagers living near the forest [6,16,24]. Communities living in rural areas commonly face inequality in health services and poorer disease outcome [4]. These vulnerable groups prefer traditional healers or wearing amulets to prevent malaria rather than seeking treatment at healthcare centres [28]. The community perspective and preventive behaviour of malaria can also be influenced by local beliefs on disease aetiology [28]. In Indonesia, it is believed that the disease is caused by supernatural causation [28]. Poor knowledge and different perspectives about disease aetiology increases the vulnerability because the individual tends to perceive the risk poorly and not practise the appropriate preventive measures [28]. Understanding this crucial context of the local community belief and how it influences the behavioural intentions, including cultural approval or disapproval of certain behaviours, may help explain a wide range of health preventive behaviours to the community and predict their compliance [29,30,31].

In addition, some communities also perceived *P. knowlesi* malaria as a disease of non-natural causation, thus hindering them from seeking modern treatment [28]. Worldwide, supernatural beliefs of disease causation are common [29]. Studies in Ethiopia and Indonesia reported how a group people was made vulnerable to malaria infection due to their cultural belief systems [28,29,31]. All this evidence strongly supports that, qualitative studies are needed to provide a detailed and rich description of the community belief and perspective to complement the findings from the baseline knowledge, attitude, and practice (KAP) survey in most of the health surveys [32].

A better readiness for malaria infection can be supported by high awareness of the disease with regard to the knowledge about the disease transmission within the community and also community participation in disease control [15,33]. Direct community participation plays a major role in malaria preventive measures, besides the top-down intervention that is initiated by policymakers and other agencies [15,33]. Furthermore, a close understanding of their social context and belief plays a major role too. Many vector-borne diseases are part of the complex ecological system and any unintended impact on non-target organisms should be avoided [33]. In theory, the individuals’ belief, attitude, cultural norm, and other social context may influence their behaviour [34]. However, various other external factors can also influence human behaviour towards disease prevention [14,15]. An individual’s sociocultural belief would determine their choice of treatment [32]. For instance, a mother may delay seeking treatment for her sick child due to her belief that her child is having an ordinary fever instead of malaria. Thinking that the illness is not life-threatening, they prefer to “wait-and-watch” [35]. Therefore, it is vital to understand the local belief. Issues such poor and unclear communication between healthcare workers and community members can reduce the sensitivity of communities in recognising malaria symptoms. Cultural knowledge, perceived severity of illness, and past experiences with the illness are also major factors to be considered in malaria prevention [35]. No communities should be stigmatised for their cultural beliefs or blamed for the high prevalence of infections among them [32].

In establishing guidelines, scientific knowledge should be complemented with close attention to sociocultural implications, behavioural practices, and the community views on the prescribed measures. Currently, exploratory studies on *P. knowlesi* malaria exposure in the SEA are lacking. Only limited published studies are available from the Philippines and Indonesia [28,30]. An intervention that is designed without a clear understanding of the underlying human behaviour and theories would be less effective [32]. Exploratory studies provide logic and reasoning that can expand and provide more depth of the discovery [32]. The views from the community can facilitate a sustainable malaria programme [33]. Therefore, more exploratory studies on *P. knowlesi* exposure should be conducted to obtain more comprehensive information about the communities and populations that are vulnerable to this infection.

### 1.9. Human Behaviour

The majority of published studies in determining the risk factors of *P. knowlesi* infection focused on the sociodemographic, socioeconomic, and environmental factors, as well as the protective nature of genetic factors such as G6PD enzyme deficiency. However, the detection of infection, including asymptomatic infection among all age groups and gender requires the identification of risk factors that are related to human behaviours [6,15]. It is important to characterise individual behaviours that predispose to a higher risk of *P. knowlesi* infection because of the complex nature of the infection. The transmission occurs via a person who is bitten by the mosquito vectors harbouring the *Plasmodium* parasite. A study by Grigg et al., in Sabah, Malaysia outlined significant individual risk factors to *P. knowlesi* malaria and recommended future studies to conduct in depth exploration of human behavioural factors [6]. Various ethnicities living in the Malaysian Borneo Island are highly exposed to this zoonotic malaria because their livelihoods are primarily based on forest-related activities such as hunting and agricultural activities [5]. The occurrence of peri-domestic transmission highlighted the dynamic changes in the epidemiology of *P. knowlesi* malaria [26]. Furthermore, the occurrence of *P. knowlesi* malaria across different age groups and settings requires the identification of when and where the exposure is occurring, especially the characteristic of night-time activities that put the vulnerable communities at risk. While insecticide-treated-bednets (ITNs), long lasting insecticide-treated nets (LLINs), and insecticide residual spraying (IRSs) are effective to control human malaria traditionally, they are somehow less effective in preventing *P. knowlesi* infection [16]. This can be attributed to the distinctive behaviour of the vector of *P. knowlesi* malaria that belongs to the *Leucosphyrus* group of *Anopheles* mosquitoes, that are forest-dwelling mosquitoes [27]. Thus, specific intervention targeted at *P. knowlesi* malaria is needed.

Behavioural change is one of the key determinants of a successful malaria intervention [15,33]. A better understanding of behaviour and human activities in a certain population can provide deeper views from the communities’ perspective and their social norms, on more feasible and suitable malaria preventive measures [15,36]. Various factors influence human behaviour. Poor compliance with the preventive measures may increase the probability of acquiring malaria [28]. For instance, the consistent usage of ITNs was proven to significantly reduce malaria incidence by 50.0% and the under-five mortality [4]. However, the effectiveness of ITNs could only be achieved with consistent usage every night [4,14,33]. Apart from that, *Anopheles* mosquitoes are exophagic biters, thus putting the communities that perform regular outdoor or forest-related activities at a higher risk of mosquito bites [16]. Personal protective measures such as the use of insecticide repellent and clothing are effective in avoiding outdoor mosquito bites [33]. Although these methods have proven effective, a lack of clear understanding and compliance of the usage and compliance can compromise their effectiveness [14,15]. Besides the importance of understanding the transmission dynamics of zoonotic malaria, the human behaviour of adhering and complying to the preventive strategies is also critical to guide the effective intervention strategies, especially in customising the strategies to target specific places, groups, and activities.

**Figure 2:**
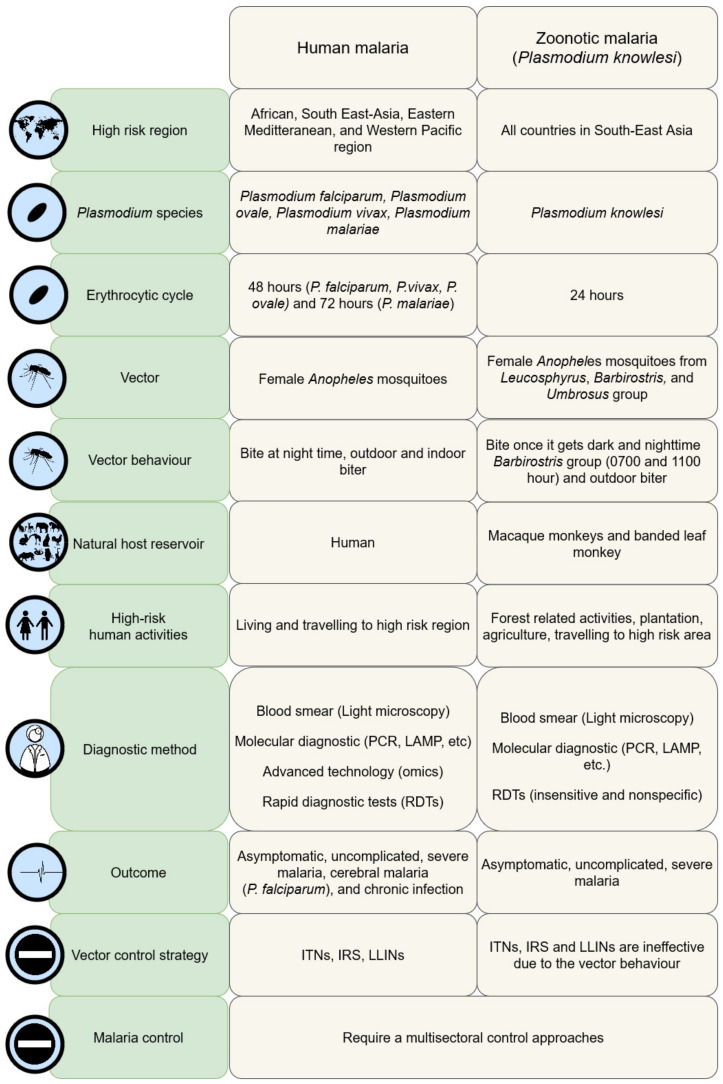
The comparison of human malaria and *P. knowlesi* malaria.

Moving toward the target of global malaria elimination, the biggest challenge lies with the difficulty in controlling and/or eliminating zoonotic malaria due to the disproportionate impact of zoonotic malaria on public health as compared to human malaria. It is increasingly crucial to understand human behaviours based on the local context, because the vulnerable communities with a high risk to *P. knowlesi* malaria infection have different lifestyles, social norms, and activities in the epidemiological context [15]. As such, prevention and control programmes should be tailored to the targeted groups by identifying their interests and customising the incentives to guarantee a higher level of success [33]. Behavioural modifications must be made practical and simple, in addition to the provision of tools that are affordable and accessible to encourage these communities to switch to positive attitudes. The sustainability and success of the malaria control programme rely on community participation for long term behavioural maintenance rather than a one-time trial of practice [33]. Moreover, due to the complexity of the epidemiology of *P. knowlesi* malaria, community participation in the zoonotic malaria intervention programme may be impractical without the support and encouragement of the multi-sectoral agencies [15]. In short, community participation and behavioural change are powerful components in changing the social norms among vulnerable communities that are exposed to *P. knowlesi* infection (see Figure 2).

### 1.10. Theories and Models to Support the Concept Paper

The exploration of various theories and models is crucial to support the concept of this paper. Theories can guide future studies and serve as the theoretical lens for researchers [32]. It can also provide a systematic explanation of a diverse range of social phenomena [37]. Globally, there are many different perspectives to a phenomenon or research question [37]. Thus, the researcher may be interested in either testing the theory (confirmatory study), generating a new theory (exploratory study), or both. However, neither of these have been optimally employed in the research continuum and evolving research question regarding *P. knowlesi* malaria disease. Exploratory research among the human population does not only look at the social determinants and societal conditions that can cause the disease, it also incorporates biomedical and scientific investigation of the disease to identify the most diagnostic, treatment, prevention, and control strategies [32]. Kamat [35] explored how community belief of disease and illness might influence the treatment-seeking behaviour for malaria disease. Storey et al. [38] examined the Ideation Model, a metatheory model to underpin the identification of potentially effective malaria preventive measures in Mali, Madagascar, and Nigeria. 

The following subtopics elaborate on the relevant theories that should be integrated in the future study of *P. knowlesi* malaria. A critical approach that is practised by social scientists is the need to focus the attention on both the formal healthcare science and informal practices that are part of the social norm and community lifestyle [32]. With the improved understanding of the complexity surrounding the vulnerability to *P. knowlesi* malaria infection, factors that are associated with the social dimension should be viewed closely with other technical and biomedical factors.

### 1.11. Social Determinant of Health Framework

The Social Determinants of Health (SDH) Framework is frequently used across studies in the healthcare sector, especially research that evaluates how social inequalities and injustices compromise ’people’s health. The SDH framework assists the researcher to describe a complex condition where people are born, grow, live, work, and age [39]. This framework consists of both upstream and downstream factors and processes that interact dynamically and in a non-linear way over an individual’s lifetime.

According to this framework, social inequity and social injustice can be determined by examining the patient’s socioeconomic position (SEP) such as age, education, occupation, income, and political context (governance, policies, culture, and societal values) [39]. The above-mentioned factors can be the root cause of health and health equity. The SDH also highlights the behavioural, environmental (physical and social), and biological factors as the intermediate determinants [39]. Hence, most preventive efforts should focus on the intermediary level that involves the community, and not just be confined to the proximal level that focuses on the politics to indirectly determine the health and its outcomes.

### 1.12. Theory of Planned Behaviour

The Theory of Planned Behaviour (TPB) is a psychological theory that links behaviour to individual belief [34]. Attitude, subjective norms, and perceived behaviour control are important components in the prediction of the behavioural intention of respondents using this theory. The theory also posits that behavioural intentions immediately determine behaviours and under certain circumstances, are directly by perceived behavioural control [34].

### 1.13. Social Cognitive Theory

The Social Cognitive Theory (SCT) emphasises the triadic reciprocal determinism in which it highlights that an individual’s personal factors, behaviours, and environmental factors interact and influence one other bidirectionally [40]. This theory suggests that each factor exerts different strengths and weaknesses in influencing a person’s behaviour and not all occur simultaneously. For instance, the goals, expectations, beliefs, self-perception, and intention play a significant role in designing and shaping the behaviour of an individual [40]. However, individuals may evoke different behaviours from their social environment. In comparison, TPB has been shown to have a larger effect in determining the behaviour intention of individuals than SCT, but more research is warranted [41]

### 1.14. The Protection Motivation Theory

The Protection Motivation Theory (PMT) is a psychological theory that describes how individuals are motivated to react to fear appeals by threat appraisal and coping appraisal [42]. These triggers include fear messages that encourage individuals to take protective measures or to refrain from activities that might harm themselves or others. Self-efficacy is argued to be the most powerful predictor of human behaviour intentions that influence the actual behaviour of an individual. A robust self-efficacy is more likely to manifest as protective behaviour and to induce the likelihood of taking effective remedial action [42].

### 1.15. The Ideation Model

This model has been used by the Social Behaviour Change (SBC) campaign. It conceptualises that effective communication can influence cognitive, social support, and emotion at the individual or community level, towards socio and behaviour change [43]. The “ideation” is a new way of thinking. It is based on local context and takes into account various factors such as beliefs and values, social norms, emotional responses, as well as social support [38,43]. The model consists of psychosocial variables that play a role in determining the intention and behaviour by reinforcing and confirming decisions. Human malaria infection is related to predictors surrounding the usage of bednets including the quantity of bednet availability, age of the users, household size, regions, and others [38]. While there was a positive relationship between the ideation of female caregivers and the household’s bednet usage, it was definitely not a liner relationship. The behaviour is more complex and involves many causal pathways [38]. Due to the cumulative effect of ideation variables across different populations, health messages should be delivered through well-integrated communication strategies [38]. More importantly, this model suggests that communication and other social context play an influential role in the behavioural change towards malaria infection [38,43]. Towards the improvement of *P. knowlesi* malaria control strategies, researches should consider to explore the ideation factors of the vulnerable population that are affected by this infection.

### 1.16. Murdock’s Model: The Aetiology of Illness (1978)

The model recognises the critical role of indigenous ill-causal beliefs when describing the local belief on disease causation [44]. The illness is believed to be caused by either natural or supernatural factors. It conceptualises the beliefs about ill-health causation within the study community, especially on how they perceive the causes of illness. For generations, the society has lived and evolved, producing a cultural “evolution” that gradually adapted certain views on the health problems of the society through trial-and-error behaviours [44]. This anthropological view believes the concept of primitive medicine as a substantial component of pragmatic knowledge. For example, quinine, a component of cinchona trees that has been used culturally as an antimalarial treatment, has been incorporated as part of modern medicine to treat malaria [45]. In addition, the theory on illness causation also postulates that certain communities perceive that diseases such as malaria can be related to supernatural, natural, and societal causes [15,29]. Based on the sociocultural perspective of the community living in the North East Ethiopia, by tapping into the unique perspective of this community, local evidence can be gathered and analysed as a foundation towards implementing more effective and suitable localised healthcare strategies [29]. At times, experience and norms living in the community, influence a person’s belief to the causes of *P. knowlesi* malaria and effective preventive measures [15]. Therefore, policymakers are recommended to incorporate community belief about illness causation in the development and implementation of public health programmes for the purpose of health promotion and disease prevention [15,29].

### 1.17. The Cytokine Theory 

According to research, the concentration of cytokines can be influenced by the malaria parasites in the blood vessels [21]. This theory suggested that the sequestered parasites in blood vessels, i.e., schizogony, led to higher local concentrations of cytokines compared to the average level within the blood circulation. Furthermore, the products of schizogony of the *Plasmodium* could trigger the release of cytokines such as Tumour Necrosis Factor (TNF) and Interleukin-1 (IL-l) [21]. The serum levels of these cytokines correlated with illnesses such as cerebral symptoms in human malaria. Furthermore, the administration of these cytokines can cause malaria-like symptoms such as altered mental status [21].

### 1.18. The Explanatory Model

The explanatory model (EM) facilitates researchers in understanding and demonstrating the influences of sociocultural variations among the patients and their close contacts (e.g., friends, relatives, or particular social groups) in contrast to the etic of medical practitioners [32,45]. Interventions using EM as a research tool enable the researcher to explore the local understanding of illness in terms of disease aetiology, experiences, diagnosis, and treatment-seeking behaviours of illnesses, as well as the community structures that are supportive towards disease prevention [46]. EM can also be used to explain the distinction between disease or illness, cultural iatrogenesis, and its core adaptation, all of which have practical implications for clinical, public health, and research. For example, the question arises when only one person is infected with malaria despite being in the same place at the same time with other people [45]. The person may question why he/she is the only one who is infected and others remain well [45]. To design a well-accepted community programme, a close alliance between the public health practitioners and the community members is vital to obtain a clear understanding and expectations of the community. Effective malaria programmes should take into account all the deeply rooted local sociocultural reasonings that guide the people’s behaviour.

In the EM, five inquiries often guide researchers, namely (i) aetiology, (ii) onset of symptoms, (iii) pathophysiology, (iv) course of sickness, and (v) treatment, can be elicited from the emic or perspective of patients and close persons to them during a particular episode of sickness should be inquired [46].

Additionally, the EM concept emphasises the role of communication in delineating the conflicts on illness construct between the professional and layman [32,46]. For instance, what people believe and experience when they are ill are usually far more complex and deeply interconnected with their daily lives. These questions can guide the researcher on how to explore the vast notions of disease among different individuals and groups or populations so that suitable preventive action as well as effective and timely treatment can be planned.

1. How can you get malaria infection?
2. Why do you think you have been exposed to malaria infection?
3. Why is malaria infection serious?
4. How are your symptoms before you were diagnosed with malaria? [prompt: Do they get worst?]
5. If you have re-infection, how serious can it get?
6. Why is early treatment necessary?
7. How to prevent malaria infection?

## 2. Discussion

After the discovery of a large focus of *P. knowlesi* infection in 2004 among humans [1], researchers have expanded their scopes of investigation to include more aspects of *P. knowlesi* malaria [2,3,5,15,16,33,34]. Following the dynamic changes in the epidemiology of *P. knowlesi* malaria infection, the identification of this *Plasmodium* species and factors that are associated with *P. knowlesi* malaria exposure should take into account the socio and behavioural context of the vulnerable population. Other relevant immune factors such as cytokine levels should be included as a prognostic factor to predict the outcome of the disease, as highlighted in previous study that explored the vulnerability factors of *P. knowlesi* malaria [20].

From the public health perspective, new strategies for *P. knowlesi* detection and prevention are paramount now that the infection is more prevalent among many different sociodemographic groups. The emerging threat of *P. knowlesi* infection in the human population requires urgent planning for early recognition and timely management [18]. While rapid diagnostic tests (RDTs) are beneficial for the fast detection of human malaria, it is less sensitive and specific for *P. knowlesi* malaria [3]. Other new point-of-care diagnostic methods such as using loop-mediated isothermal amplification (LAMP), a nucleic acid amplification technique, can accurately diagnose malaria infection rapidly and accurately [47]. Furthermore, LAMP is a simple diagnostic method where a person can directly visualise the result with naked eyes, unlike the tedious and costly PCR [47]. A simple water bath with isothermal condition is needed for the procedure [47]. LAMP has been recommended as a possible substitute for the molecular diagnosis of *P. knowlesi* malaria [48]. Despite all the recent advances, the application of these technologies remains constrained, especially as many malaria endemic regions are developing countries with limited resources [4,49].

In addition, the SES also predispose individuals to *P. knowlesi* malaria infection. A recent review highlighted the important roles of sociodemographic, SES, and environmental factors to *P. knowlesi* malaria [2,5,15]. The evidence of poverty (e.g., housing structures, rural location, occupation) is an important determinant of health outcome that puts the vulnerable population at risk for malaria exposure [25]. With regard to this, however, other components from the social context, belief, human behaviour, and immune mediators such as cytokine levels are equally paramount in the development of a context-specific framework that is useful for research. The detection of asymptomatic *P. knowlesi* cases requires a deeper understanding of human immunity. Further research should focus on the immunity responses such as the cytokine levels during malaria infection, as highlighted by previous studies [20,22]. More research should be performed on immunity response, such as cytokine levels as highlighted by Janet C.S et al. [20] and other studies on human malaria infection [22].

Nevertheless, most of the relevant published studies were quantitative in nature. An in-depth qualitative method can provide more evidence to obtain a holistic picture of the *P. knowlesi* disease exposure [15]. The rich description from qualitative studies at its natural settings, often produces rich information that can assist in the design of alternative preventive measures that are more suitable to the local settings [45]. It is vital to venture further beyond a simplified, standardised tool for *P. knowlesi* malaria control towards more enhanced approaches that are customised for different population groups, ages, and areas. The bottom-up approach via community engagement in malaria control can provide more sustainable and effective strategies as compared to the top-down vertical method [33].

Although all these factors have been discussed in previous studies, the vulnerability factors of *P. knowlesi* malaria infection warrant further exploration to determine the “logic” behind all the human-related factors. While existing studies on *P. knowlesi* malaria emphasised the role of sociodemographic factors, SES, and environmental factors as the predisposing factors of human infection, future research should be expanded to obtain a deeper understanding of the roles of social context and human behaviour on disease exposure. A further finding between poverty for example, to *P. knowlesi* malaria exposure is not sufficient by a superficial documentation on housing structures and their access to interventions. Many questions remain on how current intervention suits their activities and social norms in avoiding the mosquitoes’ bites, thus addressing this inequalities issue should be prioritised in future studies. An in-depth understanding of human behaviour, especially the interaction between humans and mosquitoes is imperative to mitigate disease transmission. The human context as a vulnerable factor of *P. knowlesi* exposure should not be neglected. The role of social scientists in revealing the complexity and uncertainties of *P. knowlesi* malaria exposure among the vulnerable communities should be re-visited to identify and address any gaps in the prevention and control strategy of *P. knowlesi* malaria. A paradigm shift is urgently needed to avoid “rigor mortis” in the epidemiological aspect of infectious diseases research [32]. The exploratory study will lead to a better understanding of the community-related strategies that are critically needed for more feasible and successful *P. knowlesi* malaria control.

**Figure 3:**
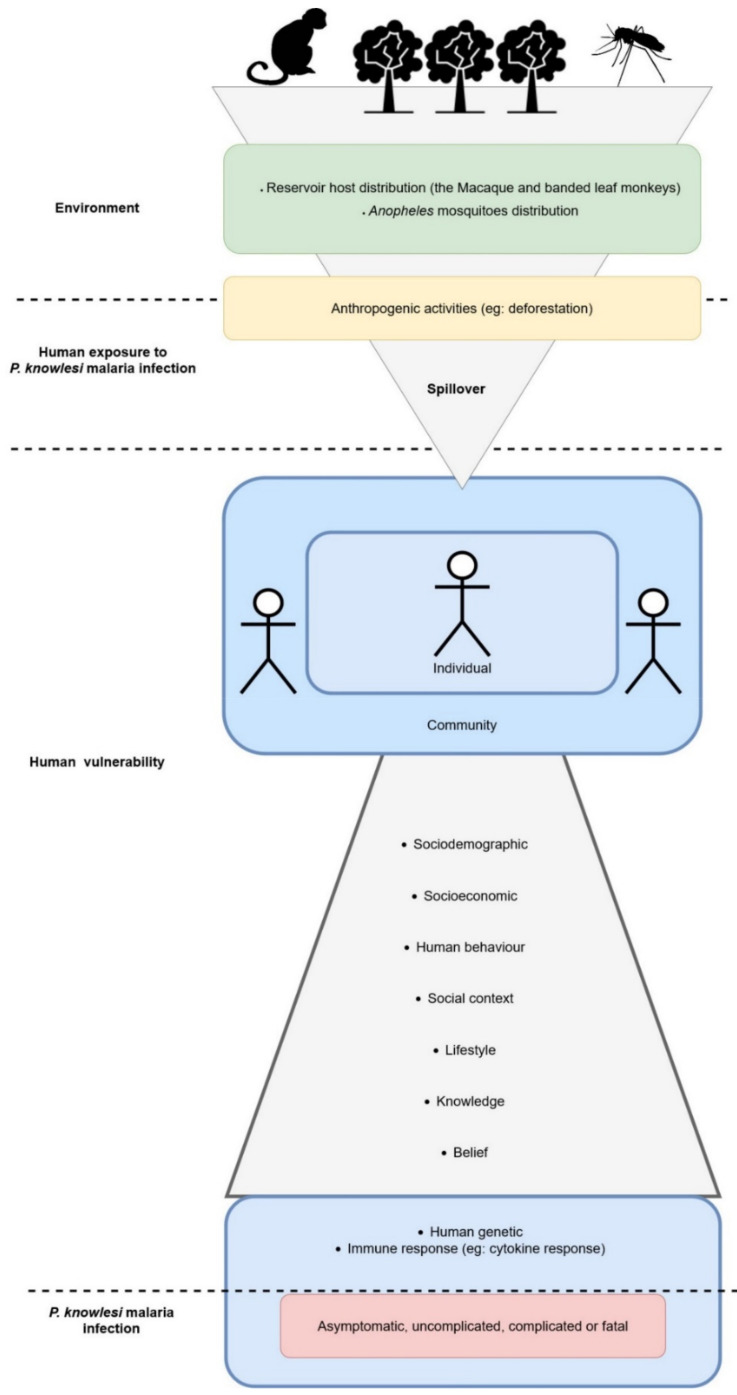
The vulnerable factors of *P. knowlesi* malaria infection in the human population.

We propose a concept that is holistic to encompass various factors that can play bigger roles in providing more effective strategies for *P. knowlesi* malaria control among vulnerable communities (Figure 3). This would help future researchers to examine factors that influence the infection of *P. knowlesi* malaria in humans, from various perspectives.

## 3. Conclusions

Current evidence indicates that the incidence of *P. knowlesi* malaria infection is increasing across SEA countries. More worryingly is the detection of asymptomatic *P. knowlesi* infection that calls for a paradigm shift in *P. knowlesi* malaria control. For asymptomatic malaria, research on the immune response that focuses on cytokines can provide the necessary information to differentiate between asymptomatic and symptomatic patients. Apart from the quantifiable epidemiological factors and cytokine levels, social determinants are also essential in achieving a more robust assessment of the vulnerability factors to *P. knowlesi* malaria infection. Nevertheless, the existing studies appear to be contentious and the evidence from these studies showed mixed results about human-related factors. Local beliefs and perceptions of the signs and symptoms of malaria infection often contradict biomedical evidence. Besides issues on social inequalities, the community at risk needs to continue living and performing their norms, and thus, tends to persist with activities that continue to expose them to greater exposure and risk to *P. knowlesi* malaria infection, despite the control strategies that are put in place. As such, the combat against this zoonotic disease remains a challenging task that requires a huge multi-sectoral commitment. With the increasing prevalence of asymptomatic *P. knowlesi* cases in humans, future approaches must be tailored to mitigate the disease transmission among the targeted vulnerable communities to reduce the emerging threat. Historically, exploratory studies are often side-lined compared to scientific studies. This paper proposes a concept to tap into the social context and human behaviour among the vulnerable communities to obtain a deeper understanding of zoonotic malaria exposure in association with the sociodemographic, SES, and environmental risk factors of the communities. The proposed concept can guide future research regarding human-related factors of *P. knowlesi* malaria infection. More importantly, it will be useful in the planning of studies that are relevant to the evaluation of the *P. knowlesi* malaria control programme as well as to guide policymakers on the development and implementation of targeted interventions to reduce the future disease burden.

## 4. Study Strengths and Limitations

This study integrated various vulnerable factors of *P. knowlesi* in humans. The evidence highlighted that various factors can potentially lead to a paradigm shift in *P. knowlesi* malaria infection and its control, especially from the perspective of social context and human behaviours of the vulnerable communities. The recommendation to design *P. knowlesi* control strategies using the bottom-up approach is valid considering the challenges in eliminating the infection due to the presence of its natural hosts (the *Macaque* monkeys, banded leaf monkeys, and *Anopheles* mosquitoes), and the increasing prevalence of asymptomatic *P. knowlesi* cases in the population.

The main limitation of this study was the main focus on *P. knowlesi* malaria alone. Thus, the findings cannot be generated for other human malaria (e.g., *P. falciparum*). Future studies should strive to identify any overlapping features between *P. knowlesi* malaria with other malaria infections such as *P. falciparum* and *P. vivax* malaria. There is also a lack of studies concerning the role of human genetics (e.g., haemoglobin variants), and immunity studies (e.g., cross-immunity) on the susceptibility of humans to the complex zoonotic *P. knowlesi* infection. While these are important contexts to consider, they lie outside the scope of this study. Future studies on cross-protective immunity can be performed to explain the phenomenon. Despite this limitation, this paper includes vital points for future study references, especially with regard to the various behavioural theories that can be adapted for future studies, considering the importance of behavioural change at the community level to ensure the sustainability of *P. knowlesi* malaria intervention.

## Data Availability

The data for this study are available upon request to the corresponding author.
